# Prediction of Positive Angiogram Using Enhanced Computed Tomography in Postpartum Hemorrhage: A Retrospective Multicentered Study

**DOI:** 10.7759/cureus.81221

**Published:** 2025-03-26

**Authors:** Makoto Aoki, Hiroyuki Tokue, Hisao Yajima, Masazumi Tani, Terutaka Yoshihara

**Affiliations:** 1 Advanced Medical Emergency Department, Critical Care Center, Maebashi Red Cross Hospital, Maebashi, JPN; 2 Traumatology Department, National Defense Medical College Research Institute, Tokorozawa, JPN; 3 Diagnostic Radiology and Nuclear Medicine Department, Graduate School of Medicine, Gunma University, Maebashi, JPN; 4 Emergency and Critical Care Medicine Department, SUBARU Health Insurance Society Ota Memorial Hospital, Ota, JPN

**Keywords:** angiography, computed tomography, embolization, postpartum hemorrhage, prediction

## Abstract

Aim: This study aimed to determine the accuracy of active arterial hemorrhage (AAH) on computed tomography (CT) in predicting the need for embolization in postpartum hemorrhage (PPH).

Methods: In a multicentered retrospective observational study, we reviewed the medical records of PPH patients between April 2010 and May 2020. We included patients who initially underwent enhanced CT and were subsequently classified as AAH+ or AAH- on CT. AAH+ on angiogram and embolization was used as a positive reference standard. A multiple logistic regression model evaluated AAH on CT as a predictor for embolization. We assessed AAH+ on CT supplied by extrauterine arteries.

Results: Of 231 PPH patients, 94 underwent enhanced CT. AAH+ and AAH- on CT consisted of 44 and 50 patients, respectively. Of AAH+ on CT patients, 32 (72.7%) underwent angiography and embolization. The sensitivity, specificity, and positive and negative predictive values of AAH on CT were 95.6%, 69.0%, 50.0%, and 98.0%, respectively. A multiple logistic regression model revealed that AAH on CT was an independent predictor of embolization (odds ratio=9.31, confidence interval=2.85-30.50, p<0.01). Sites of AAH on CT were the vagina (n=7), intraperitoneal (n=3), and abdominal wall (n=3), except for intrauterine sites.

Conclusion: Enhanced CT showed high sensitivity and had a negative predictive value for predicting AAH+ on angiogram and embolization; this may be useful in ruling out the necessity for angiography. Active arterial hemorrhage on CT may predict embolization and identify the precise bleeding site.

## Introduction

Postpartum hemorrhage (PPH) is a life-threatening condition that is the most important cause of maternal death worldwide [1,2]. A delay in diagnosis and appropriate treatment for PPH is associated with maternal death [3,4]. Therefore, the immediate detection and localization of the bleeding site is vital for rescuing patients with severe PPH [5].

Uterine artery embolization (UAE) has been the standard treatment option for PPH because of its low invasiveness and high success rate [6,7]. However, the following two unresolved problems exist with UAE: first, which patients with PPH should undergo angiography is still debated. Angiography is indicated for patients with PPH who are unresponsive to initial management and in whom specific causes of PPH, such as uterine atony-resistant uterotonics, cervico-uterine hemorrhage, vaginal thrombus or inaccessible to any surgical procedure; however, an accurate and appropriate indication was undetermined [8,9]. Second, PPH may involve active arterial bleeding, except for uterine arteries, and conventional angiography may yield limited information regarding extrauterine bleeding from ovarian and inferior epigastric arteries, as well as the peripheral branches of the internal iliac artery [10,11].

Recently, enhanced computed tomography (CT) was shown to be useful in identifying candidates for angiography and detecting the location of postpartum hemorrhage [8,12-14]. Active arterial hemorrhage (AAH) on CT was an indicator for UAE and the site of the AAH determined which artery needed to be embolized. While negative angiograms may occur in patients with AAH on CT, and because UAE has defined complications, further information is needed as to when angiography should follow the recognition of AAH on CT in patients with PPH.

How often AAH on CT will yield a positive angiogram and the need for embolization is lacking in the literature. Therefore, we evaluated the predictivity of AAH on CT for a positive angiogram and embolization in patients with PPH. Our hypothesis was that AAH on CT had a high sensitivity and positive predictive value; however, the specificity and negative predictive value may be low. Patients with PPH have to undergo UAE because of the other aforementioned clinical characteristics, and we additionally developed a multiple regression model to assess the independent effect of AAH on CT for embolization.

## Materials and methods

Study design and patient selection

A multicentered, retrospective, observational study was performed. All methods were performed in accordance with the Strengthening the Reporting of Observational Studies in Epidemiology (STROBE) guidelines. Three emergency and perinatal medical centers contributed retrospectively and collected clinical imaging data. All participating hospitals had similar availabilities of CT-scanning and interventional radiology, and similar qualities of patient treatment. The study protocol was approved by the Institutional Review Board of Gunma University Hospital (#HS2021-058), with informed consent waived. Data-use agreements were executed between each institution and the host institution. The medical records of patients with PPH in these hospitals between April 2010 and May 2020 were reviewed.

Variables collected for the analysis were patient demographics, mode of conception, parity, gestational weeks at delivery, mode of delivery, types of PPH regarding interval between delivery and PPH, cause of PPH, vital signs at hospital arrival and hemodynamic instability during clinical course, laboratory results, and findings of an enhanced CT and an angiogram. Primary PPH was defined as PPH within 24 hours after delivery, and secondary PPH was defined as PPH from 24 hours to 12 weeks after delivery [8]. Hemodynamic instability was defined as systolic blood pressure <90 mmHg or shock index >1.0 during the clinical course. Coagulopathy by laboratory results was defined as a platelet count <50000/μL, prothrombin time-international normalized ratio of >2.0, or fibrinogen <150 mg/dL. Patients with PPH who underwent an initial enhanced CT examination were included. Positive findings of enhanced CT or angiogram were defined as active arterial hemorrhage (AAH) including arterial extravasation, pseudoaneurysm, or vessel interruption. Our study patients were divided into two groups according to the presence or absence of AAH on enhanced CT (AAH+ or AAH- on CT).

Multidisciplinary approach for PPH

Conventional medical treatments for PPH, including fluid resuscitation, blood transfusion, uterine massage, and administration of uterotonic drugs, were performed using a multidisciplinary approach by obstetricians, emergency physicians, anesthesiologists, and an interventional radiologist [2]. Enhanced CT examinations, angiography, and embolization were performed at the discretion of the multidisciplinary team.

Enhanced CT technique and interpretation

In the emergency department at participating hospitals, CT examinations were performed with a 64-slice multi-detector CT scanner. Dynamic phase CT was performed in the plain, arterial, and portal venous phases. Monitor scanning was initiated 10 s after the start of contrast media. Breath-hold dual-phase diagnostic scanning was performed 10 s (arterial phase) and 110 s (portal venous phase) after aortic enhancement in monitoring images that reached bolus-tracking threshold attenuation (220 HU). Active arterial hemorrhage on CT was defined as extravascular high-attenuating regions with attenuation similar to or greater than that of the aorta on arterial phase images and was analyzed by at least one radiologist. The site of AAH on CT was classified into four regions as follows: uterine body, vagina, intraperitoneal (extrauterine), and abdominal wall. Whether AAH was present on enhanced CT images was recorded for a total of four locations for each patient. Additionally, we corrected information on the visualization of the ovarian artery in the arterial phase during enhanced CT. Visualization of the ovarian artery was defined as obvious blood flow from the ovarian artery to the uterus and confirmed by two independent interventional radiologists.

Details of angiography and embolization

Angiography was performed on patients with unstable hemodynamics, AAH on enhanced CT, and/or continuous vaginal bleeding. A 5-Fr sheath was inserted into the right common femoral artery. First, the initial aortography was performed at the level of renal arteries with a 4-Fr pigtail catheter (Gifu, Japan: Terumo Clinical Supply Co. Ltd.) to identify the uterine artery and/or other potential bleeding sites. Regarding AAH+ on CT patients, we first tried to embolize any suspected bleeding artery and subsequently embolized bilateral uterine arteries if a patient’s vital signs did not improve or vaginal bleeding did not cease after embolization of the suspected bleeding artery. For AAH- on CT patients, bilateral uterine arteries were basically selected and embolized. A 5-Fr Cobra catheter (Tokyo, Japan: Medikit Co. Ltd.) was introduced over a 0.035-inch guidewire (Gifu, Japan: Terumo Clinical Supply Co., Ltd.) to assess internal iliac arteries and their branches, such as uterine arteries. Bilateral uterine arteries were selected with a 5-Fr Cobra catheter or a microcatheter (Estream 2.0; Tokyo, Japan: Toray Medical Co. Ltd.) and embolized with gelatin sponge particles (Serescue; Tokyo, Japan: Nippon Kayaku Co. Ltd.). We basically used absorbable and nonpermanent embolic materials. After completing the embolization of uterine arteries, we again performed aortography to search for other bleeding arteries such as ovarian arteries, inferior epigastric arteries, internal pudendal arteries, and vaginal arteries. Ovarian artery embolization was performed based on the physiological findings of the patient and radiological findings [2]. When the cessation of bleeding on the post-embolization angiogram and cessation of vaginal bleeding at speculum inspection, performed immediately after embolization, were confirmed, an angiography procedure was completed.

Statistical analysis

Continuous variables were reported as medians with interquartile ranges, and categorical variables were reported as counts with percentages. Basic and maternal characteristics were compared between AAH+ and AAH- on CT groups. Chi-squared and Mann-Whitney U tests were used for categorical and continuous variables, respectively. First, we calculated the sensitivity, specificity, and positive (PPV) and negative predictive values (NPV) of AAH on CT for AAH on angiogram. Embolization was set as the reference standard. Additionally, we calculated the predictivity of AAH on CT for AAH on angiogram and embolization between primary and secondary PPH. Second, a multiple logistic regression model was created to assess the independent effect of AAH on CT for embolization. Adjusted confounders were selected according to scientific rationale; age, types of PPH, and coagulopathy were selected. Third, we performed univariate analysis of outcomes between AAH+ and AAH- on CT. The following outcomes were evaluated: ovarian artery visualization on CT findings, sites of AAH on CT, sites of AAH on angiogram, quantity of blood transfusion, hospital length of stay, and hysterectomy rates. Finally, we descriptively examined embolized arteries and embolic materials between AAH+ and AAH- on CT. Statistical significance was defined as two-sided p<0.05 or assessed using a 95% confidence interval (CI). All statistical analyses were performed using R software version 4.0.5 (Vienna, Austria: R Foundation for Statistical Computing).

## Results

**Figure 1:**
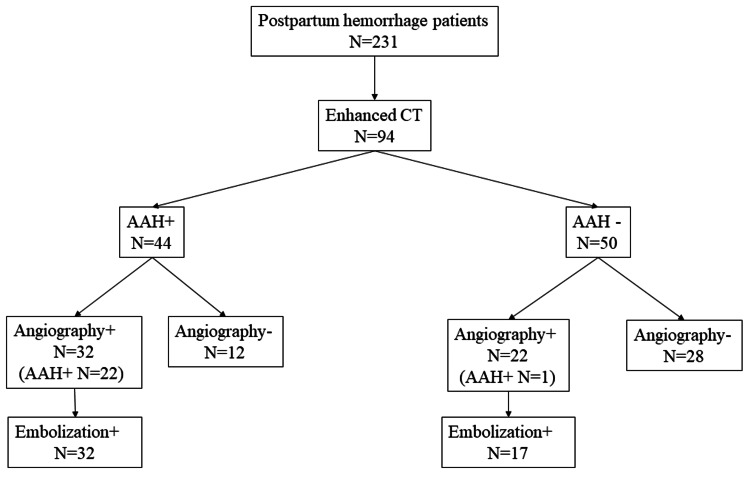
Patient clinical course and flowchart. AAH: active arterial hemorrhage; CT: computed tomography

A total of 231 patients with PPH were enrolled during the study period. Of these, 94 patients underwent dynamic enhanced CT at initial evaluation. The number of AAH+ and AAH- patients on CT was 44 and 50, respectively (Figure 1). The characteristics of these two groups are compared in Table 1.

**Table 1:** Clinical characteristics of AAH+ and AAH- patients on CT. AAH: active arterial hemorrhage; AVM: arterio-venous malformation; CT: computed tomography; HR: heart rate; IQR: interquartile range; PPH: postpartum hemorrhage; sBP: systolic blood pressure Hemodynamic instability in the clinical course was defined as sBP <90 mmHg or shock index >1. Coagulopathy was defined as a platelet count of <50000/μL, prothrombin time-international normalized ratio of >2.0, or fibrinogen <150 mg/dL.

Variables	AAH+ on CT, n=44	AAH- on CT, n=50	p-Value
Demographics			
Age, years (median)	33 (31-35)	34 (30-36)	0.96
Mode of conception, n (%)			
Spontaneous	32 (76.2)	31 (64.6)	0.25
Assisted reproductive technology	10 (23.8)	17 (35.4)	
Parity, n (%)			
Primiparous	23 (52.3)	28 (56.0)	0.83
Multiparous	21 (47.7)	22 (44.0)	
Previous normal vaginal delivery	17 (80.9)	17 (77.2)	1.00
Previous cesarean section	4 (19.0)	5 (22.7)	1.00
Gestational weeks at delivery, week, median (IQR)	39 (36-40)	39 (36-40)	0.60
Mode of delivery, n (%)			
Normal vaginal delivery	32 (72.7)	41 (82.0)	0.32
Cesarean delivery	12 (27.3)	9 (18.0)	
Interval between delivery and postpartum hemorrhage			
Primary PPH	27 (61.4)	28 (56.0)	0.67
Secondary PPH	17 (38.6)	22 (41.5)	
Cause of PPH, n (%)			
Atony	11 (25.0)	12 (25.0)	0.41
Placenta accreta	1 (2.3)	1 (2.0)	
Remnant placenta	14 (31.8)	23 (47.9)	
Placenta previa	1 (2.3)	1 (2.1)	
Trauma	7 (15.9)	7 (14.6)	
Uterus artery aneurysm or AVM	7 (15.9)	4 (8.3)	
Placental abruption	3 (6.8)	0 (0)	
Vital signs			
sBP (mmHg) median (IQR)	117 (100-135)	112 (103-125)	0.49
HR (bpm) median (IQR)	100 (86-117)	96 (86-110)	0.71
Shock index, median (IQR)	0.89 (0.68-1.10)	0.86 (0.70-1.06)	0.98
Hemodynamic instability in clinical course, n (%)	21 (47.7)	17 (34.0)	0.20
Laboratory results			
Hemoglobin values (g/dL) median (IQR)	8.2 (6.0-9.7)	8.0 (6.5-9.5)	0.81
Coagulopathy	18 (40.9)	4 (8.0)	<0.01
Fibrinogen levels (mg/dL) median (IQR)	186 (91-256)	256 (194-297)	<0.01

Spontaneous conception was 67.0% (63/94), and primiparous was 54.2% (51/94). Cesarean delivery for conception accounted for 22.3% (21/94). Primary PPH and secondary PPH were approximately 60% and 40%, respectively. Causes of PPH included the following: remnant placenta (39.3%), atony (24.4%), and trauma including vaginal or perineal laceration (14.8%), in descending order. Angiography was performed in 54 patients (57.4%) and embolization was performed in 49 patients (52.1%). Four patients required vaginal sutures for trauma and two patients underwent hysterectomy.

No significant difference between the two groups was observed in terms of the following: demographics, mode of conception, parity, mode of delivery, and cause of PPH. Additionally, vital signs on hospital arrival and the proportions of hemodynamic instability in clinical course did not significantly differ between the two groups. Regarding laboratory results, patients in the AAH+ on CT group had significantly more coagulopathy (40.9% vs. 8.0% between AAH+ and AAH- on CT), and fibrinogen values were significantly lower in the AAH+ on CT group (p<0.01). Univariate analyses of outcomes are shown in Table 2.

**Table 2:** Univariate analyses of clinical outcomes of patients with PPH who underwent initial CT evaluation (AAH+ and AAH- on CT). AAH: active arterial hemorrhage; CT: computed tomography; NA: not applicable; PPH: postpartum hemorrhage

Outcomes	AAH+ on CT, n=44	AAH- on CT, n=50	p-Value
CT findings, n (%)			
Ovarian artery visualization	18 (41.9)	11 (22.4)	0.07
Sites of AAH on CT			
Intrauterine	31 (70.4)	NA	-
Vagina	7 (15.9)	NA	-
Intraperitoneal	3 (6.8)	NA	-
Abdominal wall	3 (6.8)	NA	-
Angiography	32 (72.7)	22 (44.0)	<0.01
AAH on angiogram	22 (68.8)	1 (2.0)	<0.01
Sites of extravasation on angiogram			
Intrauterine	17 (77.3)	1 (100.0)	1.00
Vagina	2 (9.1)	0 (0)	
Intraperitoneal	2 (9.1)	0 (0)	
Abdominal wall	1 (4.5)	0 (0)	
Angioembolization	32 (72.7)	17 (34.0)	<0.01
Quantity of blood transfusion			
Red blood cells, units	8 (4-12)	4 (2-6)	0.01
Hospital length of stay	8 (6-11)	8 (6-9)	0.27
Hysterectomy	1 (2.3)	1 (2.0)	1.00

The AAH+ on CT group of patients showed significantly more AAH on angiogram (68.8% vs. 4.5%; p<0.01) and underwent significantly more embolization (72.7% vs. 34.0%; p<0.01) compared with patients of the AAH- on CT group. The sensitivity, specificity, PPV, and NPV of AAH on CT were 95.6%, 69.0%, 50.0%, and 98.0%, respectively (Table 3). Additionally, specificity and PPV were higher in secondary PPH (Table 4).

**Table 3:** Case categorization and results for assessing accuracy of AAH on CT. AAH: active arterial hemorrhage; CT: computed tomography Sensitivity=95.6%; specificity=69.0%; positive predictive value=50.0%; negative predictive value=98.0%

Variables	AAH on angiogram and required embolization	No angiogram required or negative angiogram
AAH+ on CT (n)	True positive (22)	False positive (22)
AAH- on CT (n)	False negative (1)	True negative (49)

**Table 4:** Case categorization and results for assessing accuracy of AAH on CT: comparison between primary and secondary PPH. AAH: active arterial hemorrhage; CT: computed tomography; PPH: postpartum hemorrhage Primary PPH, sensitivity=90.9%; specificity=61.3%; positive predictive value=37.0%; negative predictive value=96.4%. Secondary PPH, sensitivity=100.0%; specificity=81.4%; positive predictive value=70.5%; negative predictive value=100.0%.

Types of PPH		AAH on angiogram and required embolization	No angiogram required or negative angiogram
Primary	AAH+ on CT (n), n=27	True positive (10)	False positive (17)
	AAH- on CT (n), n=28	False negative (1)	True negative (27)
Secondary	AAH+ on CT (n), n=17	True positive (12)	False positive (5)
	AAH- on CT (n), n=22	False negative (0)	True negative (22)

A multiple logistic regression model showed secondary PPH (odds ratio {OR}=8.00; 95% CI=2.41-26.60; p<0.01), hemodynamic instability during clinical course (OR=3.75; 95% CI=1.18-11.90; p=0.02) and AAH+ on CT (OR=9.31, 95% CI=2.85-30.50, p<0.01) were independent predictors of angioembolization (Table 5).

**Table 5:** Logistic regression model of independent predictors of angioembolization. AAH: active arterial hemorrhage; CI: confidence interval; CT: computed tomography; OR: odds ratio; PPH: postpartum hemorrhage

Variables	OR	CI	p-Value
Age	1.02	0.91-1.14	0.70
Secondary PPH	8.00	2.41-26.60	<0.01
Hemodynamic instability	3.75	1.18-11.90	0.02
Coagulopathy	0.47	0.12-1.82	0.27
AAH on CT	9.31	2.85-30.50	<0.01

**Figure 2:**
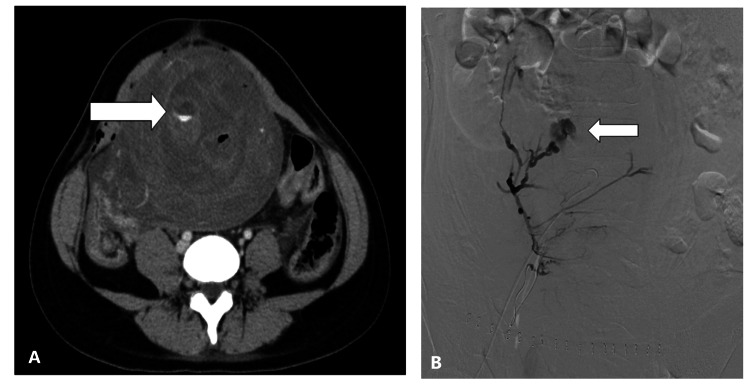
Radiologic images of a 25-year-old woman with bleeding within 24 hours after vaginal delivery. (A) A contrast-enhanced CT image shows active contrast extravasation in the uterus (arrow). (B) A right uterine arteriogram shows active contrast extravasation from the right uterine artery (arrow).

**Figure 3:**
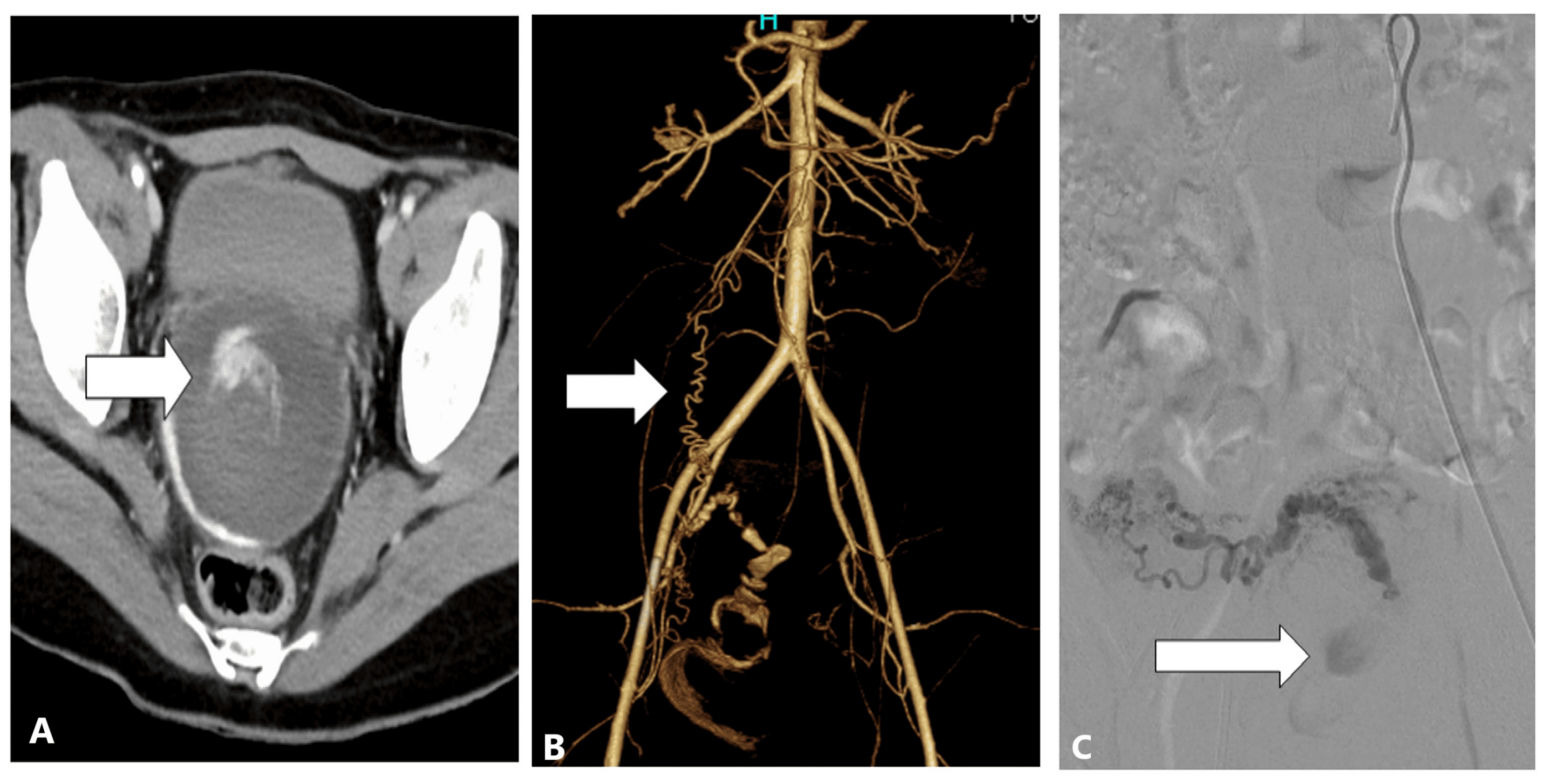
CT, CTA, and ovarian arteriogram of 23-year-old woman with postpartum bleeding within 24 hours of vaginal delivery. (A) A contrast-enhanced CT image shows active contrast extravasation in the uterus (arrow). (B) A computed tomography angiogram reveals a visible right ovarian artery (arrow). (C) A right ovarian arteriogram shows active contrast extravasation from the right ovarian artery (arrow).

**Figure 4:**
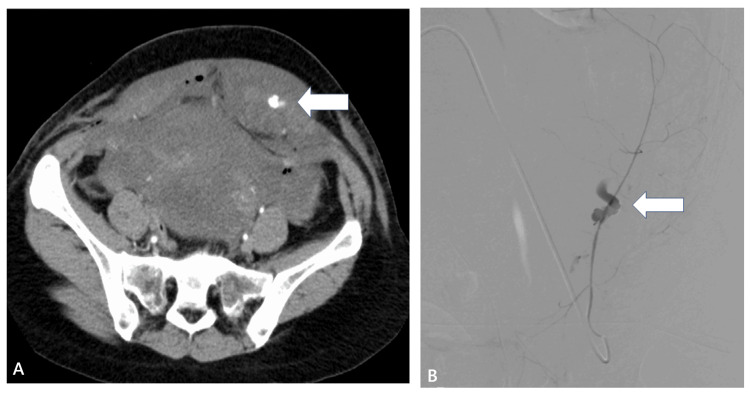
Radiologic images 38-year-old woman with bleeding within 24 hours after a cesarean delivery. (A) A contrast-enhanced CT shows active contrast extravasation in the left abdominal wall (arrow). (B) A left inferior epigastric artery angiogram shows active contrast extravasation in the abdominal wall (arrow).

The ovarian artery was more visualized on enhanced CT in the AAH+ compared with AAH- on CT group (41.9% vs. 22.4%; p=0.07). Sites of AAH on CT were the intrauterine (70.5%), vagina (15.9%), intraperitoneal location (6.8%), and abdominal wall (6.8%), respectively (Table 2, Figures 2A, 2B, 3A-3C, 4A, 4B).

AAH on angiogram was confirmed in the intrauterine (78.2%), vagina (8.6%), intraperitoneal location (8.6%), and abdominal wall (4.3%). The median quantity of red blood cells transfused was significantly higher in patients of the AAH+ on the CT group (8 {4-12} units vs. 4 {2-6} units; p=0.01). The hospital length of stay and hysterectomy rates did not significantly differ between the groups. Details of embolized arteries and embolic materials are shown in Table 6. AAH+ on CT patients tended to have other arteries embolized except for uterine arteries (31.2% vs. 11.8%; p=0.17).

**Table 6:** Details of PPH patients who underwent angioembolization: comparison between AAH+ on CT and AAH- on CT. AAH: active arterial hemorrhage; IEA: inferior epigastric artery; IIA: internal iliac artery; IPA: internal pudendal artery; NBCA: n-butyl-2-cyanoacrylate; OA: ovarian artery; UA: uterine artery; VA: vaginal artery

Variables	AAH+, n=32	AAH-, n=17	p-Value
Embolized arteries			
Uterine arteries	28 (87.5)	16 (94.1)	1.00
Bilateral UAs	25 (78.1)	16 (94.1)	0.23
Unilateral UA	3 (9.4)	0 (0)	0.54
Extrauterine arteries	10 (31.2)	2 (11.8)	0.17
OA	4 (12.5)	1 (5.9)	0.65
IPA	3 (9.4)	1 (5.9)	1.00
IIA	1 (3.1)	0 (0)	1.00
VA	1 (3.1)	0 (0)	1.00
IEA	1 (3.1)	0 (0)	1.00
Embolic materials			
Geratin	27 (87.1)	17 (100.0)	0.28
NBCA	4 (12.9)	0 (0.0)	

## Discussion

The findings from this study are as follows: (1) the sensitivity and NPV of AAH in CT group of patients were high (95.6% and 98.0%, respectively) while the specificity and PPV were low (69.0% and 50.0%, respectively). (2) A multiple logistic regression model showed that AAH on CT was an independent predictor of angioembolization. (3) Enhanced CT revealed the blood supply from extrauterine arteries. The high sensitivity and NPV of an AAH on CT were similar to previous literature [8,15].

Previous literature from Japan demonstrated high sensitivity (100%) and NPV (100%) to detect AAH on angiograms [15]. Additionally, literature from Korea showed that AAH on CT had a high sensitivity (100%) in detecting location-based AAH on angiograms. However, both studies were single-centered retrospective studies and the number of cases was small, therefore, their general validity was low.

The specificity and PPV were low compared with high sensitivity and NPV for patients in the AAH on CT group. The values of specificity and PPV in this study were also found to be similar to previous literature (specificity 28.6% and PPV 75%) [15]. On the other hand, the specificity and PPV were not so high compared with sensitivity and NPV. This was because the quality of CT examinations had improved over time, and the visualization of minor AAH on CT was enhanced [15]. Additionally, even if AAH+ on CT was confirmed, such patients did not have to undergo an angiogram and embolization. Especially in primary PPH patients, the PPV was low, and hemostasis could be achieved without embolization.

We additionally developed a multiple logistic regression model. The predictors for UAE were secondary PPH, hemodynamic instability, and AAH on CT. This result meant the significance of AAH on CT differed according to the existence of a primary or secondary PPH. The specificity and positive predictive value were higher in secondary PPH (Table 4). Regarding secondary PPH, the major causes for this were remnant placenta and a uterine artery aneurysm; the evaluation of enhanced CT may be associated with higher predictivity. Additionally, hemodynamic instability during the clinical course was one of the predictors of embolization and if the patients did not have AAH on CT, the patients may subsequently require embolization because of hemodynamic instability.

Finally, we evaluated the efficacy of enhanced CT for determining the supplying extrauterine arteries (Figure 2). Among the AAH+ on CT group of patients, more patients had supply from the ovarian arteries and tended to undergo embolization of an ovarian artery (Figure 3). AAH+ patients on CT underwent embolization of their extrauterine arteries, but this was not statistically significant (Table 6). Reports in the literature similarly described the efficacy of enhanced CT for the localization of AAH in extrauterine arteries such as ovarian arteries, vaginal arteries, or epigastric arteries (Figure 4) [8,13]. The usefulness was reported in the Cardiovascular and Interventional Radiological Society of Europe (CIRSE) standards on practice [14].

The high sensitivity of AAH on CT indicated that if a PPH patient did not have an AAH on CT, the necessity of angiogram and embolization for that patient could be ruled out. Low invasiveness was one of the features of embolization. Complications were not associated with angiography and embolization in this study, while the literature reported that angioembolization may induce procedure-related complications [16,17]. Enhanced CT obviated unnecessary angiogram and embolization, and may contribute to a decreased radiation dose and complications [12]. Considering the low specificity, the information of AAH on CT was not enough to conduct angiography and embolization, especially in primary PPH. As our logistic regression model showed, we may predict a patient requiring an angiogram and embolization by incorporating the information of AAH on CT into clinical practice.

This study has several strengths. First, this was a multicenter study that included a large number of patients giving the rarity of the use of UAE for PPH. To the best of our knowledge, this study was the largest among previous literature that assessed the accuracy of AAH on CT in predicting the need for embolization. Second, the specificity of AAH on CT was not so high and we first created the multivariable logistic regression model. The result showed that AAH on CT was an independent predictor of positive angiogram and embolization. Third, this study’s theme was a common clinical scenario, and the result of this study could be directly connected to clinical practice.

We also acknowledge several limitations to this study. First, this was a retrospective observational study that used hospital medical records of PPH patients at each hospital, and the definition of PPH was not defined in detail. The classical definition of PPH was cumulative blood loss ≥1000 mL, or bleeding associated with signs/symptoms of hypovolemia within 24 hours of the birth process [18]. Considering the definition of PPH, a few patients may not be included in this study. Second, the management of PPH may have differed between institutions. Whether PPH patients undergo enhanced CT or proceed to angiogram without CT was based on institutional protocol. All participating hospitals were emergency and perinatal medical centers, and the proportion without enhanced CT requiring angiogram was only 3.6% (5/137). Therefore, the patients suspected of continuous bleeding underwent enhanced CT, and similar qualities of treatment for PPH were assumed to be guaranteed. Third, unmeasured confounders such as the amount of blood loss and the hemodynamic status during the clinical course existed in multiple logistic regression models, and these confounders may affect the results.

## Conclusions

The sensitivity and NPV of the AAH on the CT group of patients were high, and enhanced CT may be useful for identifying PPH patients who require angiography and embolization. Additionally, enhanced CT revealed blood supply from extrauterine arteries, and AAH on CT may serve as a guide for embolization of these arteries. Incorporating AAH on CT into clinical practice may help predict which patients require angiography and embolization.
